# Mr. Shearly's Case of Abscess of the Chest

**Published:** 1805-01-01

**Authors:** W. Shearly

**Affiliations:** Member of the Royal College of Surgeons in London


### *To the Editors of the Medical and Physical Journal.*

Gentlemen,

CONSIDERING the great advantage I have myself derived from the many practical remarks contained in your valuable Journal, I think it would be highly culpable and ungrateful in me, in not giving my mite of information to my brethren, and throwing some light upon the cure of a disease which hitherto has baffled the skill of the physician and surgeon. Few, and few indeed, are the cases upon record that have been successfully treated; however, a case which I am about to mention, I trust will tend to convince the medical world, that such a disease as the Empyema is curable, especially when the constitution is in such a state as will favour the performance of an operation.

Thomas Poney, aged nineteen years, was admitted into the Royal Hospital, Deal, towards the latter end of the month of October, 1803; upon his admission he evidently laboured under all the symptoms of pneumonic inflammation, with the exception of that dull obtuse pain which the generality of patients labouring under the same disease for the most part complain. He compared the pain to a sword being run through his chest; he could not lay upon his right side, and even chose laying upon his back in preference to the left; the pulse seldom fell lower than 180, and never lower than 120; was extremely weak, and gave a sensation exactly as if the coats of the arteries were contracted. His appetite had quite forsaken him, and a little milk formed the chief part of his support. Previous to his admission, venesection had been carried to great lengths, and we were extremely careful how we employed the lancet; he expectorated mucus very freely, but at no one time could we ever detect the least signs of pus. This certainly encouraged us very much, and urged us at that time to form rather a favourable prognosis; blisters to the scrobiculus cordis and back were prescribed, which seemed to allay for a time the pain, but which, after a short period, returned with redoubled violence; chalybeates and digitalis were also administered, but without any beneficial effect. The pain gradually decreased, at last entirely left him, but the cough and hectic fever were far severer than I ever remember to have seen in a truly formed case of phthisis pulmonalis. All our hopes of cure were now given up, and the patient was evidently going to enter that undiscovered country from whose bourn no traveller returns, until, about the beginning of February, 1804, he informed us that there was a lump (using his own words) upon his left side. Upon examination the tumour actually fluctuated, and of course our minds were then made up as to the nature of the disease. Emollient poultices were immediately ordered to be applied to the tumour, and their application was continued for three or four days, the swelling evidently increasing every day in size, and becoming much softer. The operation was now thought adviseable; and the man not having the least objection, it was accordingly performed. The incision was not made where it was most adviseable that it should have been made, on account of the most depending part of the tumour being seated on the inferior part of the rib, the objection of course was the fear of wounding the intercostal artery; the incision being accordingly made upon the superior part of the ninth costa and the pleura divided, about six pints of pus flowed out. The patient's strength failing him very much, in the orifice of the wound was placed a small plug of lint, and the parts were again ordered to be poulticed. The operation having been performed in the morning, upon visiting him in the evening, and removing the poultice, and afterwards the plug, another quart came away; a febrifuge opiate was administered; and upon seeing him the following morning we found him as well, nay far better than what we expected; pected; he had passed a very good night, and the pulse had sunk at least 60, being at the time of operating 170.

Chalybeates were now given pretty freely with the addition of an antimonial bolus, and the patient was evidently mending rapidly until about a month after the operation, when on a sudden he was attacked with a violent pain in the chest, dyspnœa accompanied with a quick and rather a full pulse, and, in short, all the symptoms of inflammation seemed to have returned. Fortunately, however, these symptoms all disappeared upon his losing ten ounces of blood; there was still a very great discharge from the wound, but the matter was not so thick in consistence as at first. There was no alteration in the patient whatever for about six weeks or two months, when suddenly there appeared another abscess forming, about two inches from the wound; this was poulticed as the last had been, and was opened exactly in a similar manner; this opening was a far more desirable one than what the former one was, as being situated below it, it was of course far more dependent. The discharge from this last made orifice was more abundant than what was produced from the other one, and the patient seemed to be far more relieved by it; both wounds went on discharging, but the quantity abated every day. The patient was now able to walk about the ward, and by his own account was much more corpulent than he had been previous to his illness. It was now thought adviseable to send him home, that he might enjoy the benefit of his native air; he was accordingly invalided for that purpose, and was discharged from this hospital about six months after the operation had been performed, with only a very thin sanies oozing from the lower wound, the upper one being perfectly healed.

*Remarks upon the foregoing Case.*

It must appear obvious to every one, that the success which attended the performance of the operation was owing in a great measure to there being no organic affection of the lungs, as the patient, from his admission into the hospital until his discharge, never expectorated any purulent matter, consequently the disease must have been confined to the pleura, most probably solely to the pleura costalis. This case will also tend to shew, that where the inflammatory symptoms do not yield readily to the lancet, and that when the practitioner has the smallest doubt that the disease has terminated in suppuration, it is a duty incumbent upon him to examine very attentively the patient's thorax, to perceive if there is any swelling; and should he find that to be the case, however small that swelling may be, the sooner the operation is performed the better, as I am well convinced that many patients' lives might be saved if this plan was more generally adopted.

I am, &c.

W. SHEARLY,

Member of the Royal College of Surgeons in London.

---

